# Heterotopic Pregnancy Diagnosed with Point-of-care Ultrasound in the Emergency Department: A Case Report

**DOI:** 10.5811/cpcem.2020.2.45933

**Published:** 2020-04-23

**Authors:** Ian J. Holley, Sean P. Stickles

**Affiliations:** Washington University School of Medicine in St. Louis, Department of Emergency Medicine, St. Louis, Missouri

**Keywords:** Heterotopic pregnancy, Point-of-care ultrasound, POCUS, Emergency Medicine

## Abstract

**Introduction:**

Heterotopic pregnancies are rare. However, they are occurring with increasing frequency. Unfortunately, diagnosis is frequently delayed, with patients presenting in extremis.

**Case Report:**

We present a case of a heterotopic pregnancy diagnosed by point-of-care ultrasound (POCUS) in a woman presenting with lower abdominal pain, who had a documented normal first trimester ultrasound the day prior to presentation.

**Discussion:**

Given the increasing rates of heterotopic pregnancies, we can no longer be reassured by the presence of an intra-uterine pregnancy (IUP) in a patient with concerning signs and symptoms of a ruptured ectopic pregnancy. A thorough POCUS evaluation of the uterus and adnexa is essential for the diagnosis of heterotopic pregnancy in the emergency department.

**Conclusion:**

This case highlights the value POCUS brings to the emergency department evaluation of patients in early pregnancy.

## INTRODUCTION

A heterotopic pregnancy occurs when there is an intrauterine pregnancy (IUP) as well as an extrauterine, or ectopic, pregnancy. Traditionally, heterotopic pregnancies have been thought to be extremely rare. This was based on early theoretical calculations from 1948, which estimated the incidence of heterotopic pregnancy to be 1 in 30,000 pregnancies.^1^ However, more recent data estimates rates as high as 1:100 – 1:8000,^2^–^4^ with the highest rates occurring in patients undergoing assisted reproductive technologies (ART), such as in vitro fertilization, super ovulation, and intrauterine insemination. 3

Unfortunately, owing to the diagnostic challenges, the majority of heterotopic cases present late, with patients presenting in hemorrhagic shock or with an acute abdomen after the ectopic pregnancy ruptures.^5^ Many heterotopic pregnancies are missed on routine emergency department (ED) and obstetrics (OB) ultrasounds after visualization of an IUP.^2^–^4^,^7^ In this report we present a case of a patient diagnosed with a ruptured heterotopic pregnancy via point-of-care ultrasound (POCUS) in the ED following a documented normal IUP on routine OB ultrasound the preceding day.

## CASE REPORT

A 27-year-old female, gravida 3, para 1, at eight-weeks gestation and a remote history of treated cervicitis, presented to the ED with a two-day history of diffuse, crampy abdominal pain without vaginal bleeding or urinary symptoms. The patient had been seen in clinic the day prior to ED presentation where she underwent a transvaginal ultrasound and was documented to have a live IUP at eight-weeks gestation with normal uterus and adnexa, and a small amount of free fluid in the cul-de-sac.

In the ED she was tachycardic at 116 beats per minute and normotensive. Her abdomen was diffusely tender. A point-of-care transabdominal pelvic ultrasound was performed to evaluate the pregnancy, which noted a live IUP and left adnexal ectopic pregnancy (Image and Video) with free fluid noted in the pelvis and Morison’s pouch. OB was consulted and agreed with the diagnosis. The patient was taken emergently to the operating room and underwent a left salpingectomy, and 800 milliliters of intra-abdominal blood was evacuated. The patient was able to carry the IUP to term without further complications.

## DISCUSSION

Given the rarity of heterotopic pregnancies, the finding of an IUP on ultrasound may lead to false reassurance, resulting in missed extrauterine masses.^1^–^5^ This may explain why a majority of heterotopic pregnancies are often missed on initial ultrasound imaging.^2^–^4^,^6^ While the increased use of ART may attribute to the increased rates of ectopic pregnancies in recent years, up to one half of patients with ectopic pregnancies have no identifiable risk factors.^2^–^4^,^6^

Diagnosis of ectopic pregnancies can be difficult, and diagnosing heterotopic pregnancies are even more so. It is important that providers adopt a thorough evaluative process for women presenting with abdominal and pelvic pain in early pregnancy, with particular focus on physical exam and diagnostics, including POCUS. Physical exam findings in the setting of a ruptured extrauterine pregnancy include diffuse abdominal tenderness to light palpation or cough and cervical motion tenderness, with positive likelihood ratios of 4.2–4.5 and 4.9, respectively.^7^

Findings on ultrasound of an extrauterine pregnancy include the “blob” (extrauterine mixed echogenic mass) and “bagel” (extrauterine sac-like structure) signs in early pregnancy, fetus with measurable heart rate if presenting later,^2^,^8^ and signs of rupture, including fluid in the pelvis and Morison’s pouch. Ultrasound evaluations in early pregnancy should include evaluation in longitudinal and transverse planes throughout the uterus, as well as the adnexa. An initial evaluation using a transabdominal approach with a low-frequency curvilinear probe is appropriate, but should be followed by a transvaginal approach with a high-frequency endocavitary probe when concerning, but non-diagnostic, findings are noted.

**CPC-EM Capsule**

What do we already know about this clinical entity?

Heterotopic pregnancies are occurring with increasing frequency, and when missed result in significant morbidity for the patient.

What makes this presentation of disease reportable?

We describe a heterotopic pregnancy diagnosed on emergency department point-of-care ultrasound one day after a documented normal outpatient obstetrics ultrasound.

What is the major learning point?

Pregnant patients with abdominal pain require complete ultrasound assessment of the uterus and adnexa. The presence of intrauterine pregnancy (IUP) does not rule out an ectopic.

How might this improve emergency medicine practice?

Early identification and management of heterotopic pregnancy can provide improved outcomes for both the mother and the IUP.

Once the heterotopic pregnancy is diagnosed, treatment is surgical. Unlike in ectopic pregnancy, there is no role for methotrexate given the concomitant IUP.^9^ The surgical approach varies based on location of the ectopic, most commonly a laparoscopic approach if stable or laparotomy if unstable.^9^ As with other early-pregnancy bleeding or ectopic rupture, Rhesus negative mothers should be given Rho(D) immune globulin.

## CONCLUSION

Heterotopic pregnancy can present a diagnostic challenge in the OB clinic and ED. The increased frequency of heterotopic pregnancy, due to increased use of ART for hopeful mothers, makes it imperative that the era of “an IUP rules out an ectopic” come to an abrupt end. Providers caring for pregnant women should adopt a thorough, formalized evaluative process and consider heterotopic pregnancy in all pregnant women, particularly in those with risk factors, significant symptoms, or concerning ultrasound findings, despite the presence of an IUP.

## Supplementary Information

Video A transabdominal pelvic ultrasound demonstrating an intrauterine pregnancy (IUP) and a left adnexal mass. M-mode, which is used to determine fetal heart rate, demonstrates heart rates for the IUP and left adnexal mass of 156 beats per minute (bpm) and 158 bpm, respectively. Free fluid is visible in the right upper quadrant in Morrison’s pouch.

## Figures and Tables

**Image:**
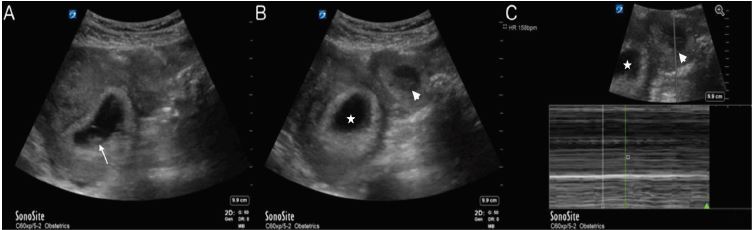
A) Visualized intrauterine pregnancy (arrow); B) Uterus (star) with ectopic pregnancy (arrowhead) seen in the left adnexa; C) Uterus (star) with M-mode through the ectopic pregnancy (arrowhead) demonstrating a fetal heart rate of 158 beats per minute.
